# The risks of adverse events with venlafaxine and mirtazapine versus ‘active placebo’, placebo, or no intervention for adults with major depressive disorder: a protocol for two separate systematic reviews with meta-analysis and Trial Sequential Analysis

**DOI:** 10.1186/s13643-023-02221-5

**Published:** 2023-03-30

**Authors:** Caroline Kamp Jørgensen, Sophie Juul, Faiza Siddiqui, Mark Abie Horowitz, Joanna Moncrieff, Klaus Munkholm, Michael Pascal Hengartner, Irving Kirsch, Christian Gluud, Janus Christian Jakobsen

**Affiliations:** 1 Copenhagen Trial Unit, Centre for Clinical Intervention Research, The Capital Region, Copenhagen University Hospital – Rigshospitalet, Blegdamsvej 9, DK-2100 Copenhagen Ø, Denmark; 2 Department of Regional Health Research, The Faculty of Health Sciences, University of Southern Denmark, J.B. Winsløws Vej 19, 3, Odense C, 5000 Odense, Denmark; 3 Stolpegaard Psychotherapy Centre, Mental Health Services in the Capital Region of Denmark, Stolpegaardsvej 28, 2820 Gentofte, Denmark; 4 Division of Psychiatry, University College London, Gower Street, London, WC1E 6BT England; 5 Research and Development Department, Goodmayes Hospital, North East London NHS Foundation Trust, Ilford, IG3 8XJ London, England; 6 Mental Health Centre Copenhagen, Copenhagen University Hospital – Mental Health Services CPH, Hovedvejen 17, 2000 Frederiksberg, Copenhagen Denmark; 7 Open Patient Data Exploratory Network (OPEN), Odense University Hospital, Odense, Denmark; 8 Department of Applied Psychology, Zurich University of Applied Sciences, Pfingstweidstrasse 96, 8005 Zurich, Switzerland; 9 Program in Placebo Studies, Harvard Medical School, 330 Brookline Avenue, Boston, MA 02215 USA

**Keywords:** Antidepressants, Venlafaxine, Mirtazapine, Major depressive disorder, Beneficial effects, Adverse events, Adverse effects

## Abstract

**Background:**

Major depressive disorder causes a great burden on patients and societies. Venlafaxine and mirtazapine are commonly prescribed as second-line treatment for patients with major depressive disorder worldwide. Previous systematic reviews have concluded that venlafaxine and mirtazapine reduce depressive symptoms, but the effects seem small and may not be important to the average patient. Moreover, previous reviews have not systematically assessed the occurrence of adverse events. Therefore, we aim to investigate the risks of adverse events with venlafaxine or mirtazapine versus ‘active placebo’, placebo, or no intervention for adults with major depressive disorder in two separate systematic reviews.

**Methods:**

This is a protocol for two systematic reviews with meta-analysis and Trial Sequential Analysis. The assessments of the effects of venlafaxine or mirtazapine will be reported in two separate reviews. The protocol is reported as recommended by Preferred Reporting Items for Systematic Reviews and Meta-Analysis Protocols, risk of bias will be assessed with the Cochrane risk-of-bias tool version 2, clinical significance will be assessed using our eight-step procedure, and the certainty of the evidence will be assessed with the Grading of Recommendations Assessment, Development and Evaluation approach. We will search for published and unpublished trials in major medical databases and trial registers. Two review authors will independently screen the results from the literature searches, extract data, and assess risk of bias. We will include published or unpublished randomised clinical trial comparing venlafaxine or mirtazapine with ‘active placebo’, placebo, or no intervention for adults with major depressive disorder. The primary outcomes will be suicides or suicide attempts, serious adverse events, and non-serious adverse events. Exploratory outcomes will include depressive symptoms, quality of life, and individual adverse events. If feasible, we will assess the intervention effects using random-effects and fixed-effect meta-analyses.

**Discussion:**

Venlafaxine and mirtazapine are frequently used as second-line treatment of major depressive disorder worldwide. There is a need for a thorough systematic review to provide the necessary background for weighing the benefits against the harms. This review will ultimately inform best practice in the treatment of major depressive disorder.

**Systematic review registration:**

PROSPERO CRD42022315395.

**Supplementary Information:**

The online version contains supplementary material available at 10.1186/s13643-023-02221-5.

## Background

### Description of the condition

Major depressive disorder is a psychiatric disorder characterised by depressed mood and diminished interest or pleasure [1]. Symptoms may include fatigue, weight loss or gain, insomnia, psychomotor agitation or retardation, feelings of worthlessness and excessive guilt, decreased concentration, and thoughts of death and suicidal ideation leading to functional and occupational impairment [1, 2]. Major depressive disorder is estimated to affect up to 264 million people globally [3, 4], making it one of the leading contributors to functional disability [3]. The high prevalence of major depressive disorder leads to an extensive economic burden estimated at more than US $210 billion annually in the United States alone, deriving from direct medical costs as well as costs related to occupational disability and comorbidities [5]. Furthermore, major depressive disorder is associated with reduced quality of life and increased risk of suicidal behaviour, with 15% of patients having attempted suicide at least once in their lifetime [6–10].

### Description of the intervention

#### Venlafaxine

Venlafaxine is often used as second-line treatment of major depressive disorder and anxiety disorders, but it is also used off-label to treat other disorders, such as obsessive–compulsive disorder, attention-deficit disorder, and migraines [11–14]. Adverse effects such as headaches, nausea, constipation, insomnia, abnormal bleeding, suicidality, and seizures have been associated with the use of venlafaxine [11, 12]. Venlafaxine is approved for the treatment of major depressive disorder in several countries, including the United States and the United Kingdom [11, 12]. Venlafaxine is classified as a serotonin–norepinephrine reuptake inhibitor (SNRI) [15]. Venlafaxine theoretically increases the synaptic levels of serotonin and norepinephrine by inhibiting their transporter proteins and, thus, blocking their presynaptic reuptake [12, 15]. This process increases the stimulation of the postsynaptic receptors, which is hypothesised to be the primary explanation of the potential antidepressant effects of venlafaxine [12, 15]. However, the role of monoamines in major depressive disorder is unclear, and the exact mechanisms of action of antidepressants, including venlafaxine, remain uncertain [16–19].

#### Mirtazapine

Mirtazapine is an atypical antidepressant commonly used as second-line treatment of major depressive disorder [14, 20]. Due to its sedative, anxiolytic, and antiemetic effects, mirtazapine is also prescribed as an off-label treatment for other psychiatric disorders, including obsessive–compulsive disorder, anxiety disorders, and post-traumatic stress disorder [20, 21]. Adverse effects such as dry mouth, sedation, increased appetite, weight gain, and suicidal ideation have been associated with the use of mirtazapine [20–22]. Mirtazapine is classified as a noradrenergic and specific serotonergic antidepressant (NaSSA) [20, 22]. Mirtazapine theoretically inhibits the presynaptic alpha 2-adrenergic receptors and the heteroreceptors on serotonergic neurons, which is hypothesised to increase the synaptic release of norepinephrine and serotonin [20, 22]. These mechanisms are thought to be related to the potential antidepressant effects of mirtazapine [22].

### Why it is important to do this review

In 2019, the total number of prescriptions in the United States were 17.71 million for venlafaxine and 6.33 million for mirtazapine [23]. Although both venlafaxine and mirtazapine are commonly prescribed to treat major depressive disorder, the balance between the beneficial and harmful effects is unclear. Previous systematic reviews have concluded that venlafaxine and mirtazapine reduce depressive symptoms with a statistically significant effect compared with placebo for patients with major depressive disorder [22, 24]. However, the effect sizes were small and may not be important to the average patient [25]. Furthermore, trials comparing antidepressants with ‘active placebo’ (a placebo that mimics the adverse effects of the experimental intervention) indicate that the beneficial effects may in fact be inflated due to the unblinding effects of the drug when compared with an inert placebo [26]. Since venlafaxine and mirtazapine have also been associated with serious adverse events, it is important to investigate whether the potential beneficial effects are outweighed by the adverse events and effects [11, 12, 20].

A network meta-analysis published in The Lancet in 2018 included placebo-controlled and head-to-head trials to assess the effects of 21 commonly used antidepressants, including venlafaxine and mirtazapine [24]. The results showed that antidepressants compared with placebo seemed to reduce depressive symptoms with a statistically significant effect (standardised mean difference (SMD) 0.30, 95% credible interval 0.26 to 0.34) [24]. The results also showed that venlafaxine and mirtazapine were some of the most effective antidepressants for reducing depressive symptoms (odds ratio (OR) 1.89, 95% credible interval 1.64 to 2.20, and OR 1.78, 95% credible interval 1.61 to 1.96) [24]. However, neither serious nor non-serious adverse events were assessed. Instead, the authors assessed lack of ‘acceptability’ (treatment discontinuation measured by the proportion of participants who withdrew for any reason) and the proportion of participants who dropped out early because of adverse effects [24]. Such data on study withdrawals as surrogate markers for harms should, however, be interpreted with caution due to difficulty attributing reasons for discontinuation, pressures on investigators to reduce the number of withdrawals, incentives of paid participants, and unblinding that often precedes decisions to withdraw [27].

Since the effects of venlafaxine and mirtazapine on depressive symptoms and quality of life have been assessed recently [24, 28], we aim to assess the risks of adverse events with venlafaxine and mirtazapine for major depressive disorder in adults including both published and unpublished data in two separate systematic reviews. Our systematic reviews will take risks of bias (systematic errors), play of chance (random errors), and certainty of the evidence into consideration. The systematic reviews will be conducted as part of a larger project investigating the beneficial and harmful effects of all antidepressants for major depressive disorder [29]. In addition to these systematic reviews, we will also publish separate systematic reviews for selective serotonin reuptake inhibitors, duloxetine [30], and tricyclic antidepressants [31]. These systematic reviews will ultimately provide data for a systematic review investigating the effects of all antidepressants for major depressive disorder [29]. This will make it possible to assess the effects of all antidepressants together, of different groups of antidepressants, and of all individual antidepressants. We chose to publish the present protocol and systematic reviews separately to investigate the effects of venlafaxine and mirtazapine in more detail (i.e. more outcomes) [29].

## Methods

The present protocol has been registered in the PROSPERO database (CRD42022315395) and is reported in accordance with the reporting guidance provided in the Preferred Reporting Items for Systematic Reviews and Meta-Analysis Protocols (PRISMA-P) statement [32, 33] (see checklist in Additional file 1).

In the following sections, we will cite the main protocol [29] when the methodology is identical.

### Criteria for considering trials for this review

#### Types of trials

‘We will include randomised clinical trials irrespective of trial design, setting, publication status, publication year, and language. We will not include quasi-randomised trials, cluster-randomised trials, or non-randomised studies.’ [29].

#### Types of participants

The participants will be adults (as defined by trialists) with a primary diagnosis of major depressive disorder as defined by standardised diagnostic criteria such as the Diagnostic and Statistical Manual of Mental Disorders, Fifth Edition [1], the International Classification of Diseases, 10th Revision [34], or earlier versions of these diagnostic manuals. ‘Major depressive disorder must be the primary diagnosis, and we will consequently not include trials randomising participants with a primary somatic diagnosis and comorbid major depressive disorder. Participants will be included irrespective of sex and comorbidities. If a trial reports data where only a subset of participants is eligible (e.g. a combination of adolescents and adults), we will only include those that fulfil our inclusion criteria, and it therefore requires that data can be obtained for that specific group’ [31].

#### Types of interventions

As experimental intervention, we will include venlafaxine or mirtazapine irrespective of dose and duration of administration. We will only include treatment arms that use doses within the licensed dose range.

As control intervention, we will include: ‘active placebo’ (a matching placebo that produces similar adverse effects to the experimental intervention, that may convince the participant and blinded outcome assessors that the participants are receiving an ‘active’ intervention), placebo, or no intervention (e.g. ‘waiting-list’).

#### Cointerventions

We will accept any co-intervention (e.g. other drug treatments or psychotherapy), if the co-intervention is planned to be delivered equally in the experimental and control groups. We will therefore also include trials where mirtazapine or venlafaxine is used as augmentation of a different antidepressant (if it is delivered in both the experimental group and the control group).

### Outcome measures

#### Primary outcomes

1. The ‘proportion of participants with either a suicide or a suicide attempt (as defined by the trialists)’ [29].
2. The proportion of participants with one or more serious adverse events. ‘We will use the International Conference on Harmonisation of technical requirements for registration of pharmaceuticals for human use—Good Clinical Practice (ICH-GCP) definition of a serious adverse event, which is any untoward medical occurrence that resulted in death, was life-threatening, required hospitalisation or prolonging of existing hospitalisation and resulted in persistent or significant disability or jeopardised the participant [35]. If the trialists do not use the ICH-GCP definition, we will include the data if the trialists use the term “serious adverse event”. If the trialists do not use the ICH-GCP definition nor use the term serious adverse event, then we will also include the data, if the event clearly fulfils the ICH-GCP definition for a serious adverse event. We will secondly assess each serious adverse event separately.’ [29].
3. The ‘proportion of participants with one or more non-serious adverse events (any adverse event not classified as serious). We will secondly assess each non-serious adverse event separately’ [29].

#### Exploratory outcomes

1. ‘Depressive symptoms measured on the 17-item or 21-item Hamilton Depression Rating Scale (HDRS) [29, 36]’. Where the 21-item scale is used, we will only include the data if the total score is only based on the first 17 items.
2. Quality of life (any valid continuous scale, e.g. the EQ-5D [37]).
3. All adverse events.
4. Individual serious adverse events.
5. Individual non-serious adverse events.
6. Suicidal ideation (any valid continuous scale assessing degree of suicidal ideation).
7. Level of functioning (any valid continuous scale).
8. ‘Depressive symptoms measured on the Montgomery-Asberg Depression Rating Scale (MADRS) [38], the Beck’s Depression Inventory (BDI) [39], or the 6-item HDRS [29, 40]’.
9. The proportion of participants with withdrawal symptoms (as defined by trialists).
10. The proportion of participants that have guessed their treatment allocation.

#### Assessment time points

Short-term follow-up will be the assessment of primary interest. Short-term follow-up will be defined as the assessment closest to 3 months after randomisation (and maximum 5 months after randomisation). Long-term follow-up, defined as 6 months or more after randomisation, will be the assessment of secondary interest.

### Search methods for identification of trials

#### Electronic searches

We will search the Cochrane Central Register of Controlled Trials (CENTRAL), Medical Literature Analysis and Retrieval System Online (MEDLINE), Excerpta Medica Database (EMBASE), Latin American and Caribbean Health Sciences Literature (LILACS), PsycINFO, Science Citation Index Expanded (SCI-EXPANDED), Social Sciences Citation Index (SSCI), Chinese Biomedical Literature Database (CBM), China Network Knowledge Information (CNKI), Chinese Science Journal Database (VIP), Wafang Database, Conference Proceedings Citation Index—Science (CPCI-S), and Conference Proceedings Citation Index—Social Science & Humanities (CPCI-SSH) to identify relevant trials. ‘We will search all databases from their inception to the present. For a detailed search strategy for all electronic databases, see Additional file 2. The search strategies for the Chinese databases will be given at review stage. Trials will be included irrespective of language, publication status, publication year, and publication type’ [29].

#### Searching other resources

We will check reference lists of relevant publications to identify other relevant randomised trials. We will email the authors of included trials to ask for unpublished randomised trials. To identify unpublished trials, we will also search clinical trial registers (ClinicalTrials.gov and the ICTRP Search Portal [41]), websites of pharmaceutical companies, websites of U.S. Food and Drug Administration (FDA), and European Medicines Agency (EMA). We will request FDA, EMA, and national medicines agencies to provide all publicly releasable information about relevant randomised clinical trials of venlafaxine and mirtazapine that were submitted for marketing authorisation, including clinical study reports [27]. ‘Additionally, we will handsearch conference abstracts from psychiatry conferences for relevant trials. We will also include unpublished and grey literature trials if we identify these, and we will assess relevant retraction statements and errata for the included trials’ [29].

### Data collection and analysis

We will perform and report the review in accordance with the recommendations stated in the Cochrane Handbook for Systematic Reviews of Interventions [27]. Analyses will be performed using Stata (StataCorp LLC, College Station, TX, USA) [42] and Trial Sequential Analysis [43, 44].

#### Selection of trials

Two review authors will independently screen titles and abstracts. The two review authors will retrieve all relevant full-text study reports/publications and independently screen the full texts and record reasons for exclusion of the ineligible trials. The two review authors will resolve any disagreement through discussion, or, if needed, they will consult with a third author.

#### Data extraction and management

Two review authors will independently extract data from included trials. Any disagreements will be resolved by discussion with a third author. The two review authors will evaluate all available data (e.g. duplicate publications, companion papers) simultaneously to maximise data extraction and correct bias assessment. We will contact the trial authors by email to obtain any additional data that may have been insufficiently reported in the publication.

#### Trial characteristics

We will extract the following data: risk of bias components (as defined below), trial design (parallel, factorial, or crossover), number of intervention groups, length of follow-up, estimation of sample size, inclusion and exclusion criteria, placebo washout period, risk of for-profit bias, registration number, and whether participants are asked to guess their treatment allocation.

#### Participant characteristics

We will extract the following data: number of randomised participants, number of analysed participants for each outcome, number of participants lost to follow-up/withdrawals/crossover, age range (mean and standard deviation), and sex ratio.

#### Intervention characteristics

We will extract the following data: dose and duration of the experimental intervention.

#### Control characteristics

We will extract the following data: type, dose, and duration of the control intervention.

#### Outcomes

All primary and exploratory outcomes will be extracted from each randomised clinical trial. We will identify if outcomes are incomplete or selectively reported according to the criteria described in the risk of bias assessment below [27].

#### Notes

We will search for information regarding industry funding of personal or academic activities for each trial author. We will judge a publication at high risk of for-profit bias if a trial is sponsored by the industry (including trials partly sponsored by the industry, e.g. if the trial drug was sponsored by a medical company), or if just one author has any affiliation to the industry. We will describe in the ‘characteristics of included studies’ table if outcome data were not reported in a usable way. Two review authors will independently transfer data into a Stata file [42]. Any disagreements will be resolved by internal discussion, or if needed, through discussion with a third author.

#### Assessment of risk of bias in the included trials

‘Our bias risk assessment will be based on the Cochrane Risk of Bias tool—version 2 (RoB 2) as recommended in the Cochrane Handbook of Systematic Reviews of Interventions [27]. We will evaluate the methodology in respect of the following bias domains:’ [29]

- Risk of bias arising from the randomisation process
- Risk of bias due to deviations from the intended interventions (effect of assignment to intervention)
- Risk of bias due to missing outcome data
- Risk of bias in measurement of the outcome
- Risk of bias in selection of the reported result

The overall assessment of risk of bias will be judged at low risk if all domains are assessed at low risk. If one or more domains are assessed at some concerns or high risk, the overall assessment will be judged at high risk.

‘We will assess the domains “missing outcome data”, “risk of bias in measurement of the outcome”, and “risk of bias in selection of the reported result” for each outcome result. Thus, we can assess the bias risk for each outcome assessed in addition to each trial. Our primary conclusions will be based on the results of our primary outcome results with overall low risk of bias. Both our primary and secondary results will be presented in the “Summary of Findings” tables.’ [29].

#### Differences between the protocol and the review

‘We will conduct the review according to this published protocol and report any deviations from it in the “differences between the protocol and the review” section of the systematic review.’ [29]

#### Measurement of treatment effect

##### Dichotomous outcomes

‘We will calculate risk ratios (RRs) with 95% confidence interval (CI) for dichotomous outcomes, as well as the Trial Sequential Analysis-adjusted CIs’ [29].

##### Continuous outcomes

‘We will calculate the mean differences (MDs) and consider calculating the SMD with 95% CI for continuous outcomes. We will also calculate Trial Sequential Analysis-adjusted CIs’ [29].

#### Dealing with missing data

We will use intention-to-treat data if provided by the trialists [45]. As the first option, we will contact all trial authors to request any relevant missing data for data extraction or assessment of risk of bias.

##### Dichotomous outcomes

We will not impute missing values for any outcomes in our primary analysis. However, we will impute data in our sensitivity analyses (see below).

##### Continuous outcomes

‘We will primarily analyse scores assessed at single time points. If only changes from baseline scores are reported, we will analyse the results together with follow-up scores [27, 29]’. If standard deviations (SDs) are not reported, we will, when possible, calculate the SDs using trial data. We will use intention-to-treat data where possible. We will not impute missing values for any outcomes in our primary analysis. However, we will impute data in our sensitivity analysis for continuous outcomes (see below).

#### Assessment of heterogeneity

‘We will primarily investigate forest plots to visually assess any sign of heterogeneity. We will secondly assess the presence of statistical heterogeneity by chi^2^ test (threshold P < 0.10) and measure the quantities of heterogeneity by the I^2^ statistic [46, 47]. We will investigate potential heterogeneity through subgroup analyses’ [29]. We may ultimately decide that a meta-analysis should be avoided due to heterogeneity [27].

#### Assessment of reporting biases

We will use funnel plots to assess reporting bias if ten or more trials are included in a meta-analysis. We will visually inspect funnel plots to assess the risk of small trial effects that could potentially reflect publication bias. We are aware of the limitations of a funnel plot (i.e. a funnel plot assesses bias due to small sample size). From this information, we will assess possible risk of publication bias. ‘For dichotomous outcomes, we will test asymmetry with the Harbord test [48] if τ^2^ is less than 0.1 and with the Rücker test if τ^2^ is more than 0.1. For continuous outcomes, we will use the regression asymmetry test [49] and the adjusted rank correlation [29, 50]’.

#### Unit of analysis issues

We will only include randomised clinical trials. We will only use data from the first period of trials with a crossover design [27, 51]. We will only include the relevant trial arms if multiple arms are reported in a single trial. If a trial has multiple relevant experimental groups, we will divide the number of events and sample size for the control group by the number of experimental groups for dichotomous data, and keep the main score for continuous data [27]. In case of a factorial design trial, e.g. 2 × 2, the two groups receiving antidepressants will be considered experimental groups, while the two groups receiving ‘active placebo’, placebo, or no intervention will be considered control groups.

#### Data synthesis

##### Meta-analysis

We will use the statistical software Stata to analyse data [42]. We will assess the intervention effects with both random-effects model meta-analyses (Hartung–Knapp–Sidik–Jonkman) [52] and fixed-effect model meta-analyses (Mantel–Haenszel for dichotomous outcomes and inverse variance for continuous outcomes) and report both results [27, 53]. We will primarily report the most conservative results (highest *P-*value) and use the less conservative results as a sensitivity analysis [54]. We assess a total of three primary outcomes, and we will therefore consider a *P*-value of 0.025 or less as the threshold for statistical significance [54]. We will investigate possible heterogeneity through subgroup analyses. We will use our eight-step procedure to assess if the thresholds for significance are crossed [29, 54]. This eight-step procedure consists of the following steps: (1) obtain the 95% confidence intervals and the *P*-values from both fixed-effect and random-effects meta-analyses and report the most conservative results as the primary result; (2) use subgroup analyses and sensitivity analyses (see step 6) to explore the reasons for substantial statistical heterogeneity; (3) adjust the thresholds for significance according to the number of primary outcomes to take account of problems with multiplicity (we will also adjust for secondary outcomes); (4) calculate required information sizes (≈ the a priori required number of participants for a meta-analysis to be conclusive) for all outcomes and analyse each outcome with Trial Sequential Analysis. Report whether the trial sequential monitoring boundaries for benefit, harm, or futility are crossed; (5) calculate Bayes factors for all primary outcomes; (6) use subgroup analyses and sensitivity analyses to assess the potential impact of bias on the review results; (7) assess the risk of publication bias; and (8) assess the clinical significance of the statistically significant review results [54].

##### Trial Sequential Analysis

‘Traditional meta-analysis runs the risk of random errors due to sparse data and repetitive testing of accumulating data when updating reviews. We wish to control the risks of type I and type II errors’ [29]. We will perform Trial Sequential Analysis on the primary outcomes to calculate the required information size (i.e. the number of participants needed in a meta-analysis to confirm or reject a specific intervention effect) and the cumulative Z-curve’s breach of relevant trial sequential monitoring boundaries [43, 44, 55–61]. For dichotomous outcomes, we will estimate the required information size based on the observed proportion of participants with an outcome in the control group (the cumulative proportion of participants with an event in the control groups relative to all participants in the control groups), a relative risk reduction or a relative risk increase of 20%, an alpha of 2.5% for all our outcomes, a beta of 10%, and the observed diversity (which is calculated based on the available trial results [44, 54]). A more detailed description of Trial Sequential Analysis can be found in the manual [44] and at http://www.ctu.dk/tsa/.

#### Subgroup analyses

We will perform the following subgroup analyses when analysing the primary outcomes (suicides or suicides attempts, serious adverse events, and non-serious adverse events).

- Trials at high risk of bias compared to trials at low risk of bias
- Trials without for-profit bias compared to trials at unknown or known risk of for-profit bias [62]
- Type of comparator (‘active placebo’, placebo, no intervention)
- Type of definition used for serious adverse events. This may be the ICH-GCP definition, the term ‘serious adverse events’, or data that clearly fulfils the ICH-GCP definition but is not referred to by the above-mentioned definitions
- Type of diagnostic criteria (operationalised criteria versus non-operationalised criteria)
- Trials using a placebo washout period compared to trials without a placebo washout
- Trials including participants who have used antidepressants within the last 6 months compared to trials excluding participants who have used antidepressants within the last six months
- Unpublished trials versus published trials

We will use the formal test for subgroup interactions in Stata [42].

#### Sensitivity analyses

To assess the potential impact of the missing data for dichotomous outcomes, we will perform the two following sensitivity analyses on the primary outcomes:

‘Best–worst-case’ scenario: We will assume that all participants lost to follow-up in the antidepressant group had no serious adverse events, had no suicides or suicide attempts, and had no non-serious adverse events, and that all those participants lost to follow-up in the control group had a serious adverse event, died by suicide or had a suicide attempt, and had a non-serious adverse event.

‘Worst-best-case’ scenario: We will assume that all participants lost to follow-up in the antidepressant group had a serious adverse event, died by suicide or had a suicide attempt, and had a non-serious adverse event, and that all those participants lost to follow-up in the control group had no serious adverse events, had no suicides or suicide attempts, and had no non-serious adverse events.

We will present results of both scenarios in our review. To assess the potential impact of missing SDs for continuous outcomes, we will perform the following sensitivity analysis:

- ‘Where SDs are missing and it is not possible to calculate them, we will impute SDs from trials with similar populations and low risk of bias. If we find no such trials, we will impute SDs from trials with a similar population. As the final option, we will impute the mean SD from all included trials’ [29].
- ‘We will present results of this scenario in our review. Other post hoc sensitivity analyses might be warranted if unexpected clinical or statistical heterogeneity is identified during the analysis of the review results [29, 54]’.

#### Summary of findings table

We will create a summary of findings table for each primary outcome (suicides or suicide attempts, serious adverse events, and non-serious adverse events). We will use the Grading of Recommendations, Assessment, Development and Evaluations (GRADE) considerations (bias risk, heterogeneity, imprecision, indirectness, and publication bias) to assess the certainty of the body of evidence [54, 63–65]. We will assess imprecision using Trial Sequential Analysis [54], following the recommendations of Juul et al. and Korang et al. [66, 67]. We will justify all decisions to downgrade the certainty of evidence with comments in the footnotes. When interpreting our results, we will consider the impact of trial quality upon the analysis and outcomes.

## Discussion

This protocol will assess the risks of adverse events with venlafaxine and mirtazapine versus ‘active placebo’, placebo, or no intervention in two separate systematic reviews of adults with major depressive disorder. The primary outcome will be suicides or suicide attempts, serious adverse events, and non-serious adverse events.

Our protocol has several strengths. The predefined methodology is based on Cochrane methodology [27], Keus et al. [68], our eight-step assessment suggested by Jakobsen et al. [54], Trial Sequential Analysis [43], and GRADE assessment [63–65]. Thus, this protocol considers the risk of random and systematic errors as well as external validity, heterogeneity, and publication bias [54]. Moreover, we will include data from both unpublished and published trials as well as clinical study reports, which should facilitate a fairer comparison of beneficial and harmful effects [27].

Our protocol also has limitations. There are several comparisons which increases the risks of type 1 errors. We have adjusted the thresholds for significance according to the number of primary outcomes, but we have not adjusted it according to the total number of comparisons, including subgroup analyses and sensitivity analyses. We expect inadequate reporting of adverse events and effects in the included trials, which will increase the risk of underestimating harmful effects [27]. We also expect challenges with obtaining data when requesting unpublished randomised trials from regulatory authorities and pharmaceutical companies. Finally, we expect short treatment and follow-up periods which may not accurately mimic how antidepressants are used in clinical practice [69, 70].

Although venlafaxine and mirtazapine have previously been investigated in systematic reviews, the previous reviews were inadequate due to the lack of systematic assessments of adverse events and effects. Therefore, there is a need for a systematic review assessing the risks of adverse events with venlafaxine and mirtazapine compared with ‘active placebo’, placebo, or no intervention in treatment of adults with major depressive disorder. The reviews will ultimately inform best practice in the treatment of major depressive disorder.

## Supplementary Information

**Additional file 1.** PRISMA-P 2015 Checklist

**Additional file 2.** Search strategies for ‘Venlafaxine or Mirtazapine for major depressive disorder’ Preliminary search strategies prepared 4 March 2022

## Data Availability

Not applicable.

## Abbreviations

BDI: Beck’s Depression Inventory
CBM: Chinese Biomedical Literature Database
CENTRAL: Cochrane Central Register of Controlled Trials
CI: Confidence interval
CNKI: China Network Knowledge Information
CPCI-S: Conference Proceedings Citation Index—Science
CPCI-SSH: Conference Proceedings Citation Index—Social Science & Humanities
EMA: European Medicines Agency
EMBASE: Excerpta Medica Database
FDA: US Food and Drug Administration
GRADE: Grading of Recommendations, Assessment, Development and Evaluation
HDRS: Hamilton Depression Rating Scale
ICH-GCP: International Conference on Harmonisation of technical requirements for registration of pharmaceuticals for human use—Good Clinical Practice
LILACS: Latin American and Caribbean Health Sciences Literature
MADRS: Montgomery-Asberg Depression Rating Scale
MDs: Mean differences
MEDLINE: Medical Literature Analysis and Retrieval System Online
OR: Odds ratio
PRISMA-P: Preferred Reporting Items for Systematic Reviews and Meta-Analysis Protocols
RoB 2: Cochrane risk-of-bias tool—version 2
RRs: Risk ratios
SCI-EXPANDED: Science Citation Index Expanded
SDs: Standard deviations
SMD: Standardised mean difference
SSCI: Social Sciences Citation Index
VIP: Chinese Science Journal Database
WHO: World Health Organization

## References

[1] American Psychiatric Association (2013). Diagnostic and statistical manual of mental disorders (DSM-5®).

[2] Pan Z, Park C, Brietzke E, Zuckerman H, Rong C, Mansur RB (2019). Cognitive impairment in major depressive disorder. CNS Spectr.

[3] World Health Organization. Depression 2020. Available from: https://www.who.int/news-room/fact-sheets/detail/depression. Accessed 1 Sept 2020.

[4] Summerfield D (2008). How scientifically valid is the knowledge base of global mental health?. BMJ.

[5] Greenberg PE, Fournier A-A, Sisitsky T, Pike CT, Kessler RC (2015). The economic burden of adults with major depressive disorder in the United States (2005 and 2010). J Clin Psychiatry.

[6] Chen Y-W, Dilsaver SC (1996). Lifetime rates of suicide attempts among subjects with bipolar and unipolar disorders relative to subjects with other axis I disorders. Biol Psychiatry.

[7] IsHak WW, Mirocha J, James D, Tobia G, Vilhauer J, Fakhry H (2015). Quality of life in major depressive disorder before/after multiple steps of treatment and one-year follow-up. Acta Psychiatr Scand.

[8] Saragoussi D, Christensen MC, Hammer-Helmich L, Rive B, Touya M, Haro JM (2018). Long-term follow-up on health-related quality of life in major depressive disorder: a 2-year European cohort study. Neuropsychiatr Dis Treat.

[9] Kessler RC, Borges G, Walters EE (1999). Prevalence of and risk factors for lifetime suicide attempts in the National Comorbidity Survey. Arch Gen Psychiatry.

[10] Qin P (2011). The impact of psychiatric illness on suicide: differences by diagnosis of disorders and by sex and age of subjects. J Psychiatr Res.

[11] National Health Service. Venlafaxine. 2018. Available from: https://www.nhs.uk/medicines/venlafaxine/. Accessed 28 Sept 2021.

[12] Singh D, Saadabadi A (2021). Venlafaxine treasure island.

[13] Malone DC (2007). A budget-impact and cost-effectiveness model for second-line treatment of major depression.. J Manag Care Pharm.

[14] NHS East and North Hertfordshire Clinical Commissioning Group, NHS Herts Valleys Clinical Commissioning Group. Guidelines on choice and selection of antidepressants for the management of depression; 2018. Available from: https://hertsvalleysccg.nhs.uk/application/files/1615/3633/3654/Guidelines_on_Choice_and_Selection_of_Antidepressants_for_the_Management_of_Depression_Final_Sept_2016.pdf . Accessed 31 Jan 2022.

[15] Suwala J, Machowska M, Wiela-Hojenska A (2019). Venlafaxine pharmacogenetics: a comprehensive review. Pharmacogenomics.

[16] Tatsumi M, Groshan K, Blakely RD, Richelson E (1997). Pharmacological profile of antidepressants and related compounds at human monoamine transporters. Eur J Pharmacol.

[17] Albert PR, Benkelfat C, Descarries L (2012). The neurobiology of depression - revisiting the serotonin hypothesis. I. Cellular and molecular mechanisms. Philos Trans R Soc Lond B Biol Sci..

[18] Warren JB (2020). The trouble with antidepressants: why the evidence overplays benefits and underplays risks - an essay by John B Warren. BMJ.

[19] Nemeroff CB (2020). The state of our understanding of the pathophysiology and optimal treatment of depression: glass half full or half empty?. Am J Psychiatry.

[20] Jilani TN, Gibbons JR, Faizy RM, Saadabadi A (2021). Mirtazapine treasure island.

[21] National Health Service. Mirtazapine. 2019. Available from: https://www.nhs.uk/medicines/mirtazapine/. Accessed 27 Sept 2021.

[22] Watanabe N, Omori IM, Nakagawa A, Cipriani A, Barbui C, Churchill R, et al. Mirtazapine versus other antidepressive agents for depression. Cochrane Database Syst Rev. 2011: 10.1002/14651858.CD006528.pub2. 10.1002/14651858.CD006528.pub2 PMC4158430 22161405

[23] Agency for Healthcare Research and Quality. Medical Expenditure Panel Survey (MEPS). 2021. Available from: https://www.ahrq.gov/data/meps.html. Accessed 28 Sept 2021.

[24] Cipriani A, Furukawa TA, Salanti G, Chaimani A, Atkinson LZ, Ogawa Y (2018). Comparative efficacy and acceptability of 21 antidepressant drugs for the acute treatment of adults with major depressive disorder: a systematic review and network meta-analysis. Lancet.

[25] Moncrieff J, Kirsch I (2015). Empirically derived criteria cast doubt on the clinical significance of antidepressant-placebo differences. Contemp Clin Trials.

[26] Moncrieff J, Wessely S, Hardy R. Active placebos versus antidepressants for depression. Cochrane Database Syst Rev. 2004;(1):CD003012. 10.1002/14651858.CD003012.pub2. 10.1002/14651858.CD003012.pub2 PMC8407353 14974002

[27] Higgins J, Thomas J, Chandler J, Cumpston M, Li T, Page M, et al. Cochrane Handbook for Systematic Reviews of Interventions: Cochrane, 2021. 2021. Available from: www.training.cochrane.org/handbook; 10.1002/14651858.ED000142 PMC10284251 31643080

[28] Stone MB, Yaseen ZS, Miller BJ, Richardville K, Kalaria SN, Kirsch I (2022). Response to acute monotherapy for major depressive disorder in randomized, placebo controlled trials submitted to the US Food and Drug Administration: individual participant data analysis. BMJ.

[29] Juul S, Siddiqui F, Barbateskovic M, Jorgensen CK, Hengartner MP, Kirsch I (2021). Beneficial and harmful effects of antidepressants versus placebo, 'active placebo', or no intervention for adults with major depressive disorder: a protocol for a systematic review of published and unpublished data with meta-analyses and trial sequential analyses. Syst Rev.

[30] Siddiqui F, Barbateskovic M, Juul S, Katakam KK, Munkholm K, Gluud C (2021). Duloxetine versus 'active' placebo, placebo or no intervention for major depressive disorder; a protocol for a systematic review of randomised clinical trials with meta-analysis and trial sequential analysis. Syst Rev.

[31] Jorgensen CK, Juul S, Siddiqui F, Barbateskovic M, Munkholm K, Hengartner MP (2021). Tricyclic antidepressants versus 'active placebo', placebo or no intervention for adults with major depressive disorder: a protocol for a systematic review with meta-analysis and Trial Sequential Analysis. Syst Rev.

[32] Moher D, Shamseer L, Clarke M, Ghersi D, Liberati A, Petticrew M (2015). Preferred reporting items for systematic review and meta-analysis protocols (PRISMA-P) 2015 statement. Syst Rev.

[33] Shamseer L, Moher D, Clarke M, Ghersi D, Liberati A, Petticrew M (2015). Preferred reporting items for systematic review and meta-analysis protocols (PRISMA-P) 2015: elaboration and explanation. BMJ.

[34] World Health Organization. International Statistical Classification of Diseases and Related Health Problems (ICD). Available from: https://www.who.int/standards/classifications/classification-of-diseases. Accessed 5 Sept 2021.

[35] (International Conference on Harmonisation of Technical Requirements for Registration of Pharmaceuticals for Human Use). ICH harmonised guideline: integrated addendum to ICH E6(R1): guideline for good clinical practice (ICH-GCP)2015; Step 2 version. Available from: https://ichgcp.net/da. Accessed 1 Sept 2020.

[36] Hamilton M (1960). A rating scale for depression. J Neurol Neurosurg Psychiatry.

[37] Brooks R (1996). EuroQol: the current state of play. Health Policy.

[38] Montgomery SA, Åsberg M (1979). A new depression scale designed to be sensitive to change. Br J Psychiatry.

[39] Beck AT, Steer RA, Brown GK (1996). Beck depression inventory-II.

[40] Timmerby N, Andersen JH, Søndergaard S, Østergaard SD, Bech P (2017). A systematic review of the clinimetric properties of the 6-item version of the Hamilton Depression Rating Scale (HAM-D6). Psychother Psychosom.

[41] ICTRP Search Portal [Internet]. 2021. Available from: https://www.who.int/clinical-trials-registry-platform/the-ictrp-search-portal. Accessed 5 May 2021.

[42] StataCorp. Stata Statistical Software: Release 16 College Station, TX: StataCorp LLC. 2019. Accessed 1 Sept 2020.

[43] Copenhagen Trial Unit. TSA - Trial Sequential Analysis [Web page]. Available from: http://www.ctu.dk/tsa/. Accessed 1 Sept 2020.

[44] Thorlund K, Engstrøm J, Wetterslev J, Brok J, Imberger G, Gluud C. User manual for trial sequential analysis (TSA) Copenhagen, Denmark: Copenhagen Trial Unit, Centre for Clinical Intervention Research. 2011. Available from: http://www.ctu.dk/tsa/files/tsa_manual.pdf. Accessed 1 Sept 2020.

[45] Jakobsen JC, Gluud C, Wetterslev J, Winkel P (2017). When and how should multiple imputation be used for handling missing data in randomised clinical trials–a practical guide with flowcharts. BMC Med Res Methodol.

[46] Higgins JP, Thompson SG (2002). Quantifying heterogeneity in a meta-analysis. Stat Med.

[47] Higgins JP, Thompson SG, Deeks JJ, Altman DG (2003). Measuring inconsistency in meta-analyses. BMJ.

[48] Harbord RM, Egger M, Sterne JA (2006). A modified test for small-study effects in meta-analyses of controlled trials with binary endpoints. Stat Med.

[49] Egger M, Smith GD, Schneider M, Minder C (1997). Bias in meta-analysis detected by a simple, graphical test. BMJ.

[50] Begg CB, Mazumdar M. Operating characteristics of a rank correlation test for publication bias. Biometrics. 1994;50(4):1088–101. 7786990

[51] Elbourne DR, Altman DG, Higgins JP, Curtin F, Worthington HV, Vail A (2002). Meta-analyses involving cross-over trials: methodological issues. Int J Epidemiol.

[52] IntHout J, Ioannidis JPA, Borm GF (2014). The Hartung-Knapp-Sidik-Jonkman method for random effects meta-analysis is straightforward and considerably outperforms the standard DerSimonian-Laird method. BMC Med Res Methodol.

[53] DeMets DL (1987). Methods for combining randomized clinical trials: strengths and limitations. Stat Med.

[54] Jakobsen JC, Wetterslev J, Winkel P, Lange T, Gluud C (2014). Thresholds for statistical and clinical significance in systematic reviews with meta-analytic methods. BMC Med Res Methodol.

[55] Wetterslev J, Thorlund K, Brok J, Gluud C (2008). Trial sequential analysis may establish when firm evidence is reached in cumulative meta-analysis. J Clin Epidemiol.

[56] Brok J, Thorlund K, Gluud C, Wetterslev J (2008). Trial sequential analysis reveals insufficient information size and potentially false positive results in many meta-analyses. J Clin Epidemiol.

[57] Brok J, Thorlund K, Wetterslev J, Gluud C (2008). Apparently conclusive meta-analyses may be inconclusive—trial sequential analysis adjustment of random error risk due to repetitive testing of accumulating data in apparently conclusive neonatal meta-analyses. Int J Epidemiol.

[58] Thorlund K, Devereaux P, Wetterslev J, Guyatt G, Ioannidis JP, Thabane L (2008). Can trial sequential monitoring boundaries reduce spurious inferences from meta-analyses?. Int J Epidemiol.

[59] Wetterslev J, Thorlund K, Brok J, Gluud C (2009). Estimating required information size by quantifying diversity in random-effects model meta-analyses. BMC Med Res Methodol.

[60] Thorlund K, Anema A, Mills E (2010). Interpreting meta-analysis according to the adequacy of sample size. An example using isoniazid chemoprophylaxis for tuberculosis in purified protein derivative negative HIV-infected individuals. J Clin Epidemiol..

[61] Imberger G, Thorlund K, Gluud C, Wetterslev J (2016). False-positive findings in Cochrane meta-analyses with and without application of trial sequential analysis: an empirical review. BMJ Open.

[62] Lundh A, Lexchin J, Mintzes B, Schroll JB, Bero L (2018). Industry sponsorship and research outcome: systematic review with meta-analysis. Intensive Care Med.

[63] Guyatt GH, Oxman AD, Vist GE, Kunz R, Falck-Ytter Y, Alonso-Coello P (2008). GRADE: an emerging consensus on rating quality of evidence and strength of recommendations. BMJ (Clinical research ed).

[64] Guyatt GH, Oxman AD, Schünemann HJ, Tugwell P, Knottnerus A (2011). GRADE guidelines: a new series of articles in the Journal of Clinical Epidemiology. J Clin Epidemiol.

[65] Schünemann HJ, Best D, Vist G, Oxman AD (2003). Letters, numbers, symbols and words: how to communicate grades of evidence and recommendations. Can Med Assoc J.

[66] Juul S, Nielsen N, Bentzer P, Veroniki AA, Thabane L, Linder A (2020). Interventions for treatment of COVID-19: a protocol for a living systematic review with network meta-analysis including individual patient data (The LIVING Project). Syst Rev.

[67] Korang SK, Juul S, Nielsen EE, Feinberg J, Siddiqui F, Ong G (2020). Vaccines to prevent COVID-19: a protocol for a living systematic review with network meta-analysis including individual patient data (The LIVING VACCINE Project). Syst Rev.

[68] Keus F, Wetterslev J, Gluud C, van Laarhoven CJ (2010). Evidence at a glance: error matrix approach for overviewing available evidence. BMC Med Res Methodol.

[69] Jakobsen JC, Katakam KK, Schou A, Hellmuth SG, Stallknecht SE, Leth-Moller K (2017). Selective serotonin reuptake inhibitors versus placebo in patients with major depressive disorder. A systematic review with meta-analysis and Trial Sequential Analysis. BMC Psychiatry..

[70] Munkholm K, Paludan-Muller AS, Boesen K (2019). Considering the methodological limitations in the evidence base of antidepressants for depression: a reanalysis of a network meta-analysis. BMJ Open.

