# Supplementary material for: The risks of adverse events with venlafaxine and mirtazapine versus ‘active placebo’, placebo, or no intervention for adults with major depressive disorder: a protocol for two separate systematic reviews with meta-analysis and Trial Sequential Analysis
Source: Syst Rev. 2023 Mar 30;12:57. doi: 10.1186/s13643-023-02221-5 (PMC10061867; doi:10.1186/s13643-023-02221-5)
Supplement: Supplementary file 2 — Additional file 2. Search strategies for ‘Venlafaxine or Mirtazapine for major depressive disorder’ Preliminary search strategies prepared 4 March 2022 [file 13643_2023_2221_MOESM2_ESM.pdf]

Search strategies for
'Venlafaxine or Mirtazapine for major depressive disorder'
Preliminary search strategies prepared 4 March 2022

Cochrane Central Register of Controlled Trials (latest issue) in the Cochrane Library (*2022, Issue 3: 691 hits*)
#1 MeSH descriptor: [Venlafaxine Hydrochloride] explode all trees
#2 MeSH descriptor: [Mirtazapine] explode all trees
#3 (venlafaxin* or ef*exor* or mirtazapin* or org*3770 or remeron*)
#4 #1 or #2 #3
#5 MeSH descriptor: [Depressive Disorder, Major] explode all trees
#6 MeSH descriptor: [Depressive Disorder] this term only
#7 MeSH descriptor: [Seasonal Affective Disorder] explode all trees
#8 MeSH descriptor: [Dysthymic Disorder] explode all trees
#9 MeSH descriptor: [Depression] explode all trees
#10 MeSH descriptor: [Affective Symptoms] this term only
#11 ((depress* or affective or dysthym*) and (disorder* or disease* or symptom*))
#12 #5 or #6 or #7 or #8 or #9 or #10 or #11
#13 #4 and #12

MEDLINE Ovid (1946 to the date of the search) (*040322: 2829 hits*)
1. exp Venlafaxine Hydrochloride/
2. exp Mirtazapine/
3. (venlafaxin* or ef*exor* or mirtazapin* or org*3770 or remeron*).mp. [mp=title, abstract, original title, name of substance word, subject heading word, floating sub-heading word, keyword heading word, organism supplementary concept word, protocol supplementary concept word, rare disease supplementary concept word, unique identifier, synonyms]
4. 1 or 2 or 3
5. exp Depressive Disorder, Major/
6. Depressive Disorder/
7. exp Seasonal Affective Disorder/
8. exp Dysthymic Disorder/
9. exp Depression/
10. Affective Symptoms/
11. ((depress* or affective or dysthym*) and (disorder* or disease* or symptom*)).mp. [mp=title, abstract, original title, name of substance word, subject heading word, floating sub-heading word, keyword heading word, organism supplementary concept word, protocol supplementary concept word, rare disease supplementary concept word, unique identifier, synonyms]
12. 5 or 6 or 7 or 8 or 9 or 10 or 11
13. 4 and 12
14. (randomized controlled trial or controlled clinical trial).pt. or clinical trials as topic.sh. or trial.ti.
15. (random* or blind* or placebo* or meta-analys*).mp. [mp=title, abstract, original title, name of substance word, subject heading word, floating sub-heading word, keyword heading word, organism supplementary concept word, protocol supplementary concept word, rare disease supplementary concept word, unique identifier, synonyms]
16. 13 and (14 or 15)
17. limit 16 to ("adolescent (13 to 18 years)" or "young adult (19 to 24 years)" or "adult (19 to 44 years)" or "young adult and adult (19-24 and 19-44)" or "middle age (45 to 64 years)" or "middle aged (45 plus years)" or "all aged (65 and over)" or "aged (80 and over)")

Embase Ovid (1974 to the date of the search) (*040322: 4564 hits*)
1. exp venlafaxine/
2. exp mirtazapine/
3. (venlafaxin* or ef*exor* or mirtazapin* or org*3770 or remeron*).mp. [mp=title, abstract, heading word, drug trade name, original title, device manufacturer, drug manufacturer, device trade name, keyword heading word, floating subheading word, candidate term word]
4. 1 or 2 or 3

5. exp major depression/
6. depression/
7. exp seasonal affective disorder/
8. exp dysthymia/
9. emotional disorder/
10. ((depress* or affective or dysthym*) and (disorder* or disease* or symptom*)).mp. [mp=title, abstract, heading word, drug trade name, original title, device manufacturer, drug manufacturer, device trade name, keyword heading word, floating subheading word, candidate term word]
11. 5 or 6 or 7 or 8 or 9 or 10
12. 4 and 11
13. Randomized controlled trial/ or Controlled clinical trial/ or trial.ti.
14. (random* or blind* or placebo* or meta-analys*).mp. [mp=title, abstract, heading word, drug trade name, original title, device manufacturer, drug manufacturer, device trade name, keyword heading word, floating subheading word, candidate term word]
15. 12 and (13 or 14)
16. limit 15 to (adult <18 to 64 years> or aged <65+ years>)

LILACS (Bireme; 1982 to the date of the search) (*040322: 39 hits*)
(venlafaxin$ or ef$exor$ or mirtazapin$ or org$3770 or remeron$) [Words] and ((depress$ or affective or dysthym$) and (disorder$ or disease$ or symptom$)) [Words]

PsycINFO (EBSCO host; 1806 to the date of the search) (*040322: 543 hits*)
S17 S15 AND S16
S16 TI adult* or Elder* or older or Geriatri* or Senil* or Old Age* or Late Life or Aged OR AB adult* or Elder* or older or Geriatri* or Senil* or Old Age* or Late Life or Aged
S15 S13 AND S14
S14 TX ((random* or blind* or placebo* or meta-analys*)) OR TI trial*
S13 S4 AND S12
S12 S5 OR S6 OR S7 OR S8 OR S9 OR S10 OR S11
S11 TX ((depress* or affective or dysthym*) and (disorder* or disease* or symptom*))
S10 MA Affective Symptoms
S9 MA Depression
S8 MA Dysthymic Disorder
S7 MA Seasonal Affective Disorder
S6 MA Depressive Disorder Expanders
S5 MA Depressive Disorder, Major
S4 S1 OR S2 OR S3
S3 TX (venlafaxin* or effexor* or efexor* or mirtazapin* or "org 3770" or org3770 or org-3770 or remeron*)
S2 MA mirtazapine
S1 MA venlafaxine

Science Citation Index Expanded (Web of Science; 1900 to the date of the search); Conference Proceedings Citation Index – Science (Web of Science; 1990 to the date of the search); Social Sciences Citation Index (Web of Science; 1956 to the date of the search), and Conference Proceedings Citation Index- Social Science & Humanities (Web of Science; 1990 to the date of the search) (*040322: 619 hits*)
#7 #5 AND #6
#6 TS=(adult* or Elder* or older or Geriatri* or Senil* or Old Age* or Late Life or Aged)
#5 #3 AND #4
#4 TI=(random* or blind* or placebo* or meta-analys* or trial*) OR TS=(random* or blind* or placebo* or meta-analys*)
#3 #2 AND #1
#2 TS=((depress* or affective or dysthym*) and (disorder* or disease* or symptom*))
#1 TS=(venlafaxin* or ef*exor* or mirtazapin* or org*3770 or remeron*)
